# Risdiplam Add‐On Therapy Following Onasemnogene Abeparvovec in Children With Spinal Muscular Atrophy and 2 *SMN2* Copies: A Multi‐Center Case Series

**DOI:** 10.1002/mus.70246

**Published:** 2026-04-11

**Authors:** Corinna Stoltenburg, Klaus Goldhahn, Arpad von Moers, Angela M. Kaindl, Claudia Weiß

**Affiliations:** ^1^ Department of Pediatric Neurology Charité–Universitätsmedizin Berlin Berlin Germany; ^2^ Center for Chronically Sick Children Charité–Universitätsmedizin Berlin Berlin Germany; ^3^ Section CNS Development and Neurologic Disease German Center for Child and Adolescent Health (DZKJ) Germany; ^4^ Department of Pediatrics and Neuropediatrics DRK Klinikum Westend Berlin Germany; ^5^ Charité – Universitätsmedizin Berlin Institute of Cell‐ and Neurobiology Berlin Germany

**Keywords:** add‐on therapy, combination therapy, onasemnogene abeparvovec, risdiplam, spinal muscular atrophy

## Abstract

**Introduction/Aims:**

Three disease‐modifying therapies are approved for individuals with spinal muscular atrophy (SMA); however, data concerning the combination of these therapies remain limited. This study aimed to evaluate the safety and efficacy of add‐on risdiplam in children who had experienced clinical deterioration despite gene therapy with onasemnogene abeparvovec.

**Methods:**

This is a retrospective case series study at two centers of children treated with risdiplam who had previously received onasemnogene abeparvovec. Therapy was evaluated by clinical examination, standardized physiotherapeutic assessments, and parent perspectives.

**Results:**

Five patients with SMA (four male and one female), diagnosed between 0 and 8 months, were included in the study. All had 2 *SMN2* copies and were started on risdiplam between five and 48 months after onasemnogene abeparvovec. Risdiplam was added due to motor regression, dysphagia, new onset of respiratory insufficiency, and/or recurrent pneumonias. Four children showed improvements in motor development, swallowing, and respiratory function. One child remained stable. Parents perceived a significant improvement in general impression, motor, and respiratory function. The add‐on therapy was well tolerated without adverse events.

**Discussion:**

Our results indicate an improvement in most children in a case series through add‐on risdiplam. Evaluating clinical outcome parameters in clinical practice may prove challenging and should be complemented by the parental perspective. The decision regarding the use of add‐on therapy in children with SMA who receive one line of treatment but show a clinical deterioration should be considered on an individual level, and assessments of predefined therapeutic goals are recommended.

**Abbreviations**
ADR: adverse drug reaction
CHOP‐INTEND: Children's Hospital of Philadelphia Infant Test of Neuromuscular Disorders
DMT: Disease‐Modifying therapy
dNIV: diurnal noninvasive ventilation
GFSS: growth‐friendly spinal surgery
HFMSE: Hammersmith Functional Motor Scale—Expanded
HINE‐2: Hammersmith Infant Neurological Examination part 2
NIV: NonInvasive ventilation
nNIV: nocturnal NonInvasive Ventilation
OA: onasemnogene abeparvovec
PEG: Percutaneous endoscopic gastrostomy
PROM: patient‐reported outcome measure
RULM: Revised Upper Limb module
SMA: spinal muscular atrophy
SMN: survival of Motor Neuron
VFSS: Video Fluoroscopic Swallow study

## 1 Introduction

Three survival motor neuron (*SMN*)‐targeted disease‐modifying therapies (DMTs) are available: An intravenous gene therapy with onasemnogene abeparvovec (OA) and two *SMN2* splicing modulators: intrathecal nusinersen and oral risdiplam [1]. The open‐label JEWELFISH trial (NCT03032172) investigated the safety and tolerability of risdiplam after treatment with other DMTs and included 14 subjects after treatment with OA. The safety profile was consistent with that of treatment‐naïve individuals, and an exploratory assessment of motor function revealed an improvement in motor function [2]. Retrospective studies have shown that adding risdiplam after OA appears beneficial [3, 4, 5, 6], although controlled clinical trial data are lacking. Consensus agreements have been reached between European and German‐Austrian‐Swiss clinical experts that a combination of two DMTs cannot be unequivocally recommended based on the current data [7, 8].

Here, we report the outcomes of five children with SMA who experienced loss of motor, respiratory, and/or bulbar function despite OA and thus received add‐on risdiplam.

## 2 Methods

This is a case series study of children with SMA who received risdiplam after OA at Charité—Universitätsmedizin Berlin or DRK Klinikum Westend, Berlin, Germany. Inclusion criteria comprised symptomatic or presymptomatic diagnosis of SMA with genetically confirmed variants in the *SMN1* gene, treatment with OA, deterioration of motor function and/or new onset of dysphagia and/or respiratory insufficiency, and add‐on risdiplam at the discretion of the treating physician. There were no specific exclusion criteria. Prospective data were collected in the disease‐specific registry SMArtCARE (Ethics Committee Freiburg‐Germany, no. EA56/18) [9]. Additional clinical and laboratory data were collected retrospectively from patient records (Ethic committee of the Charité—Universitätsmedizin Berlin, EA2/061/18). Written informed consent was obtained from all subjects and/or their legal guardians.

Treatment efficacy was assessed prior to OA, 2 months after the administration of OA, and at four‐month intervals thereafter. The eight motor development items from the Hammersmith Infant Neurological Examination part 2 (HINE‐2) were recorded by a pediatric neurologist [10], while the functional motor scores were obtained by a trained physiotherapist. The Children's Hospital of Philadelphia Infant Test of Neuromuscular Disorders (CHOP‐INTEND) was used for children under 2 years of age and those unable to sit [11]. For children above 2 years of age, the expanded version of the Hammersmith Functional Motor Scale (HFMSE) was used [12], and in those able to sit, upper limb motor function was assessed using the Revised Upper Limb Module (RULM) [13]. Caregiver interviews assessed respiratory function, complemented by polysomnography when indicated. Indicators of respiratory deterioration included infections and hospitalizations, with reduced hospital stays defined as a therapeutic goal. The gold standard for evaluating swallowing function is the longitudinal videofluoroscopic swallow study (VFSS) [14]. As this was unavailable for some patients, we assessed weight‐for‐age *z*‐scores (ped(z) calculator [15]) as an indirect indicator of possible dysphagia [16] and also obtained perspective interviews from caregivers and assessment results from pediatric neurologists and speech therapists. Patient‐reported outcome measures (PROMs) were collected from caregivers. A modified Clinical Global Impression of Change Scale [17] was used, with a 5‐level Likert scale ranging from much improved to much worse compared to 3 months before.

Adverse drug reactions (ADRs) associated with risdiplam were evaluated at two and 4 months after risdiplam initiation, and then every 4 months, through medical history and physical examinations. In addition, laboratory tests were ordered at the physician's discretion.

## 3 Results

**FIGURE 1:**
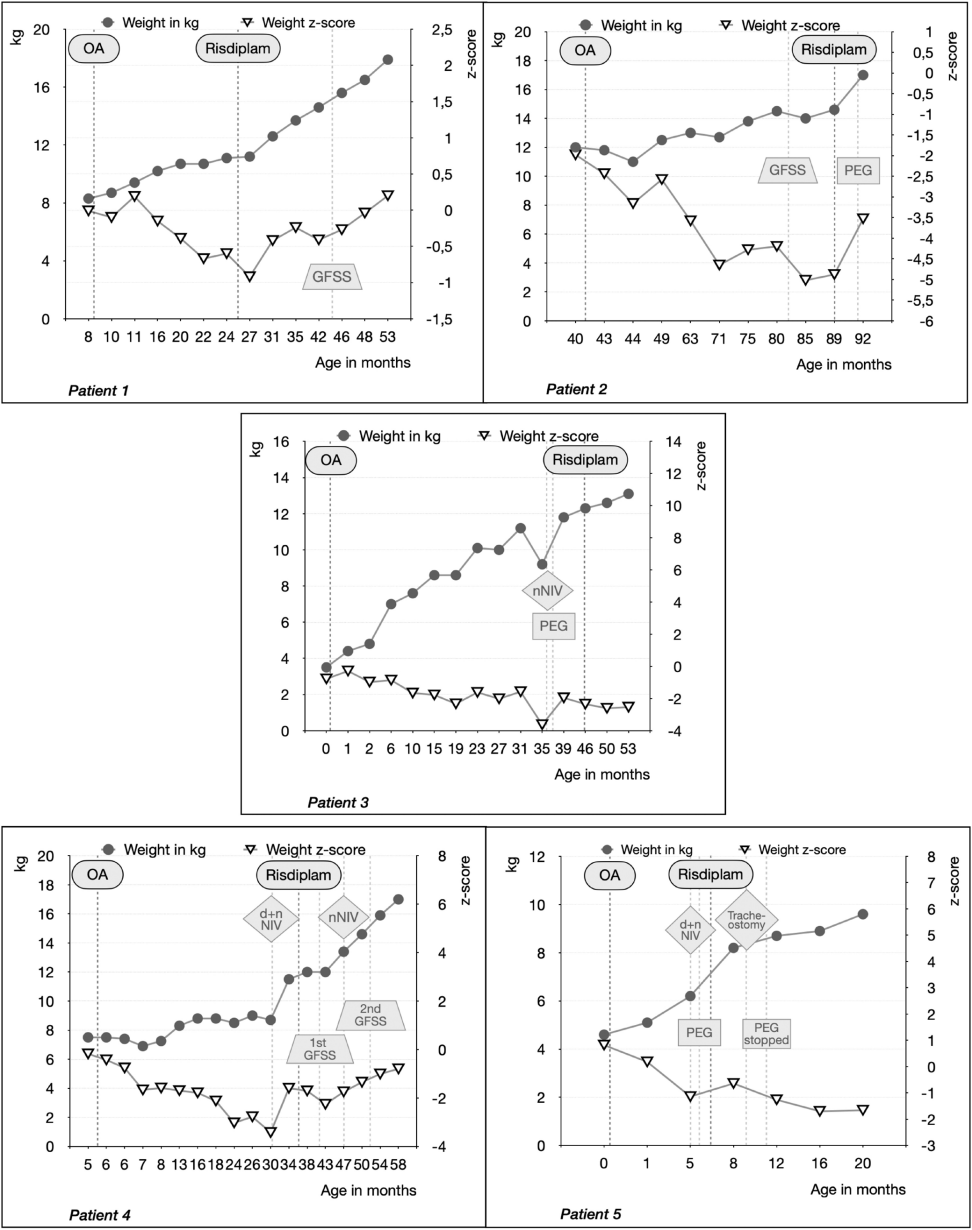
Weight‐for‐age *z*‐score and clinical course. The weight‐for‐age *z*‐score is presented in relation to the time of gene therapy, the start of add‐on therapy with risdiplam, and important clinical events such as orthopedic surgery, the placement of a gastrostomy, the start of ventilation, and tracheostomy. Abbreviations: dNIV: Diurnal noninvasive ventilation; GFSS: Growth‐friendly spinal surgery; nNIV: Nocturnal noninvasive ventilation; NIV: Noninvasive ventilation; PEG: Percutaneous endoscopic gastrostomy; OA: Onasemnogene abeparvovec.

**FIGURE 2:**
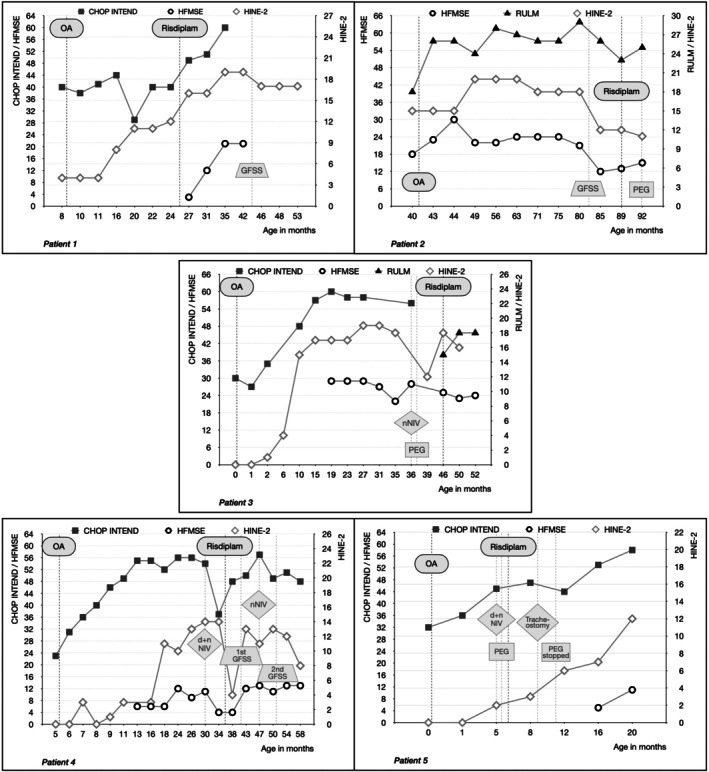
Functional scores and clinical course. The functional scores CHOP‐INTEND, HFMSE, RULM, and HINE‐2 are presented in relation to the time of gene therapy, the start of add‐on therapy with risdiplam, and important clinical events such as orthopedic surgery, the placement of a gastrostomy, the start of ventilation, and tracheostomy. In the natural course, the CHOP‐INTEND score declines by 1.71 points per month in SMA patients with 2 *SMN2* copies and neonatal onset [18]. Abbreviations: CHOP‐INTEND: Children's Hospital of Philadelphia Infant Test of Neuromuscular Disorders; dNIV: Diurnal noninvasive ventilation; GFSS: Growth‐friendly spinal surgery; HFMSE: Hammersmith Functional Motor Scale Expanded; HINE‐2: Hammersmith Infant Neurological Examination part 2; nNIV: Nocturnal noninvasive ventilation; NIV: Noninvasive ventilation; OA: Onasemnogene abeparvovec; PEG: Percutaneous endoscopic gastrostomy; RULM: Revised Upper Limb Module.

**FIGURE 3:**
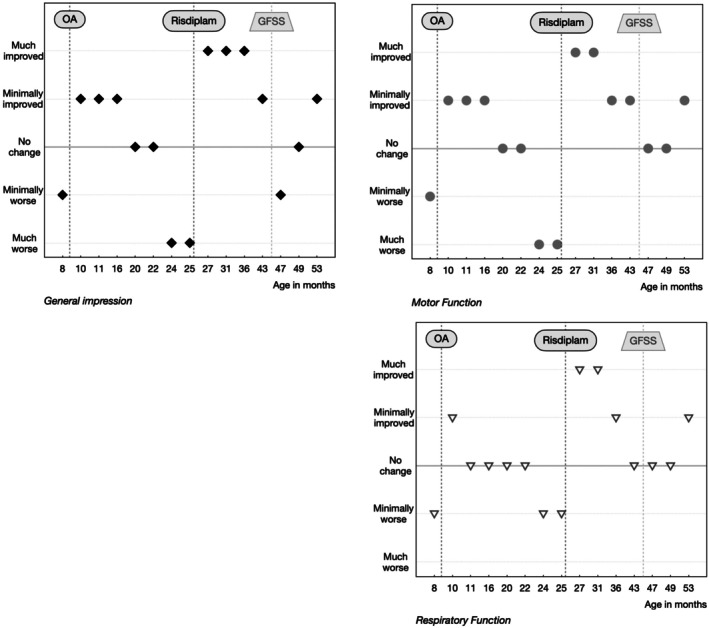
Patient‐reported outcome measures (PROMs) and clinical course in patient 1. General (a), motor (b), and respiratory (c) PROMs as reported by parents are presented in relation to the time of gene therapy, the start of add‐on therapy with risdiplam and orthopedic surgery. Each time point reflects the perceived change compared to 3 months before. Abbreviations: GFSS; Growth‐friendly spinal surgery; OA, Onasemnogene abeparvovec.

Five children with SMA met the inclusion criteria, four of whom were male. All had two *SMN2* copies and symptom onset within the first month. They had been diagnosed between 0 and 8 months of age, one through newborn screening and one through prenatal diagnosis. OA had been administered between 0 and 42 months. The subjects were started on risdiplam between 5 and 48 months after OA after they experienced a clinical deterioration of motor, respiratory and/or bulbar function (Table 1). After add‐on treatment, weight remained stable (*n* = 1) or increased (*n* = 3) in 4/5 children. One of the children who gained weight had a previously planned PEG tube placement. The only child with a slight weight‐for‐age *z*‐score decrease was a subject in whom the feeding situation had improved and subsequently the tube feeding had been discontinued (Figure 1). Permanent noninvasive ventilation (NIV) could be discontinued in patient 4, and patients 2, 4, and 5 had fewer hospitalizations for respiratory infections (Supporting Information results, Table 1). HINE‐2 scores increased by 5 and 9 points for two children, decreased by 1 and 2 points for two children and decreased by 6 points for one child who needed two spinal surgeries during follow‐up. CHOP‐INTEND increased by 20, 13, and 11 points in the three children for whom it was recorded. HFMSE increased by 18, 3, 6, and 9 points in four children and decreased by 1 point in one child. RULM increased by 2 and 3 points in the two children for whom it was assessed (Figure 2). PROMs revealed a significant perceived improvement following add‐on therapy (Figure 3). No safety issues were recorded. For individual courses refer to Supporting Information results.

**TABLE 1:** Baseline characteristics and clinical data of patients who received add‐on therapy with risdiplam.

		Patient 1	Patient 2	Patient 3	Patient 4	Patient 5
Sex		Female	Male	Male	Male	Male
Age at symptom onset (months)		~1	< 1	< 1	< 1	< 1
Age at diagnosis (months)		8	1	< 1	5	< 1
Symptoms at diagnosis		Muscular hypotonia, unable to sit, poor head control, paradoxical breathing, tongue fasciculations	Respiratory insufficiency, muscular hypotonia	Prenatal diagnosis, muscular hypotonia, weakness of lower limbs at 2 weeks of age	Muscular hypotonia, proximal weakness, lack of head control	Presymptomatic (newborn screening), muscular hypotonia, reduced spontaneous movements, muscular weakness at 3 weeks of age
DMT before OA		n.a.	Nusinersen	n.a.	n.a.	n.a.
Age at dosing of OA (months)		8	42	< 1	6	< 1
Reason for add‐on risdiplam		Motor regression Dysphagia	Respiratory insufficiency Dysphagia Motor regression	Respiratory insufficiency Dysphagia Motor regression	Respiratory insufficiency Motor regression	Respiratory insufficiency Frequent pneumonia Dysphagia Motor regression
Age at start of risdiplam (months)		25	90	46	36	6
Interval between OA & Risdiplam (months)		17	48	45	30	5
Adverse drug reactions after start of risdiplam		no	no	no	no	no
Age at last evaluation (months)		55	99	56	62	20
Respiratory support; respiratory infections	Pre risdiplam	Insufflator/exsufflator, daily use	Nocturnal NIV, start at 1 month‐of‐age; Insufflator/exsufflator; recurrent pneumonia	Nocturnal NIV, start at 36 months of age; Insufflator/exsufflator; pneumonia	Permanent NIV, start at 30 months of age; recurrent pneumonia	Nocturnal NIV, start at 5 months of age; recurrent pneumonia
	Post risdiplam	Insufflator/exsufflator, occasional use	Nocturnal NIV, occasional insufflator/exsufflator, no more pneumonia	Nocturnal NIV; pneumonia	Nocturnal‐only NIV; two pneumonia	Invasive ventilation, start at 9 months of age no more hospitalizations for respiratory infections
Nutritional support	Pre risdiplam	Fully oral; chewing and swallowing anormal	Fully oral; chewing and swallowing with difficulties Indication for PEG *(postponed due to respiratory insufficiency)*	PEG, start at 36 months‐of‐age	Fully oral	PEG, start at 6 months of age
	Post risdiplam	Fully oral; can bite and chew better; no more choking	PEG, start at 92 months of age; improved swallowing	PEG	Fully oral	Fully oral, PEG stopped at 11 months of age

Abbreviations: DMT = disease‐modifying treatment; n.a. = not applicable; NIV = noninvasive ventilation; PEG = percutaneous endoscopic gastrostomy.

## 4 Discussion

Children who are treated with OA usually maintain motor milestones, respiratory, and bulbar function [19, 20, 21]. However, children with two or three *SMN2* copies are at risk of developing significant deficits in motor and bulbar function despite early treatment and require close monitoring [20, 21, 22, 23, 24, 25, 26]. Add‐on risdiplam treatment was beneficial in five children with severe clinical deterioration in motor, respiratory, and/or bulbar function after OA.

While add‐on therapy is used more frequently in the USA, it is rarely used in Germany [8]. This explains why most children in a multi‐center retrospective case series reported by Svoboda et al. had a lack or plateau of motor improvement before add‐on therapy while all children in our case series experienced motor regression before risdiplam [3]. Similarly, dysphagia (60% vs. 40%) and ventilator dependence (80% vs. 50%) were more frequent in our cohort. Following the implementation of add‐on therapy, many children of the US cohort and all children of our cohort exhibited motor improvements. Of the 20 children in the US, 7 showed improvement in dysphagia and 6 in respiratory function [3], whereas the majority of our patients exhibited bulbar and respiratory improvements. Both case series demonstrate a positive outcome for children with inadequate improvement after OA, with children who have experienced clinical deterioration benefiting particularly from add‐on therapy. In contrast, preemptive add‐on *SMN2* augmentation therapy with risdiplam and/or nusinersen was well tolerated but improved motor development only minimally in seven children with two *SMN2* copies [22]. The ongoing open‐label studies HINALEA 1 and 2 aim to evaluate effectiveness and safety of risdiplam after OA in children < 2 years (NCT05861986 and NCT05861999).

As a limitation of our study, we encountered difficulties in objectifying motor deterioration. Motor function is typically evaluated using the motor function tests employed in pivotal trials for the DMTs. However, the scores are influenced by the compliance of the affected child. Additionally, after orthopedic spinal or hip surgery, tasks such as lying down or sitting up cannot be performed, so that a child will automatically lose scoring points despite no actual loss of gross motor force.

Another limitation is that evaluation of respiratory function is not always straightforward in young children who are not yet able to perform a lung function test. Sleep studies impose a considerable burden on families and can only be conducted at longer intervals in a routine setting. Respiratory infections and hospitalizations were used as substitute criteria, but these do not necessarily reflect poor lung function or a weak cough. Nevertheless, the reduction of respiratory infections and shorter ventilation time may indicate improved respiratory function. The evaluation of dysphagia was limited by the lack of VFSS in some children. Weight was assessed as a surrogate parameter. Even if children with SMA without dysphagia often do not have an age‐appropriate weight due to muscular atrophy, increased respiratory caloric demand, and fatigue, a longitudinal increase of the weight‐for‐age *z*‐score may indicate improved endurance of the chewing and swallowing muscles.

In addition to attempting to objectify changes in motor, respiratory, and bulbar function, the parental perspective on the disease trajectory of their children should also be considered [27]. Accordingly, the decision to initiate add‐on therapy and the evaluation of a therapeutic effect were also informed by the results of numerical PROMs on general, motor, and respiratory function as well as individually reported changes. A more detailed assessment of subjective health status using generic and disease‐specific PROMs of functional limitations, pain, and fatigue could be helpful for future treatment decisions and evaluations. However, a pronounced positive perception can also indicate a placebo effect.

The low number of patients, the wide range of age at diagnosis, at age of treatment with OA and add‐on risdiplam represent further limitations to this study.

In conclusion, the administration of add‐on risdiplam has the potential to enhance the condition of children diagnosed with SMA who have experienced a deterioration in motor, respiratory, and/or bulbar function following gene therapy with OA. The decision should be based on a comprehensive evaluation of each individual case. It is advisable to set individual treatment goals prior to the initiation of add‐on therapy and to evaluate these over the course of treatment.

## Author Contributions

**Arpad von Moers:** writing – review and editing, investigation, resources, conceptualization. **Klaus Goldhahn:** writing – review and editing, investigation. **Angela M. Kaindl:** supervision, writing – review and editing, resources, conceptualization. **Claudia Weiß:** conceptualization, writing – original draft, investigation, methodology. **Corinna Stoltenburg:** conceptualization, formal analysis, writing – review and editing, visualization, investigation, methodology.

## Ethics Statement

We confirm that we have read the Journal's position on issues involved in ethical publication and affirm that this report is consistent with those guidelines.

## Conflicts of Interest

C.W. is principal investigator of the Hinalea trial at Charité—Universitätsmedizin Berlin and has served as a paid consultant for Roche, Novartis and Biogen. A.M.K. is an advisory board member of Desitin and Stoke, and has received funding from Neuraxpharm, Angelini Pharma, Vertex, and Jazz Pharmaceuticals. The remaining authors have no conflicts of interest.

## Supporting information


**Data S1:** mus70246‐sup‐0001‐supinfo.docx.

## Data Availability

The data that supports the findings of this study are available in the Supporting Information of this article.
