# Supplementary material for: Risdiplam Add‐On Therapy Following Onasemnogene Abeparvovec in Children With Spinal Muscular Atrophy and 2 SMN2 Copies: A Multi‐Center Case Series
Source: Muscle Nerve. 2026 Apr 11;73(6):1164–71. doi: 10.1002/mus.70246 (PMC13138358; doi:10.1002/mus.70246)
Supplement: Supplementary file 1 — Data S1: mus70246‐sup‐0001‐supinfo.docx. [file MUS-73-1164-s001.docx]

***Supplemental results: Individual case reports***

***Patient 1***

Following gene therapy with OA, the girl achieved head control. At 22 months, parents noted a significant decline in the child's endurance and muscle strength (**Figure 3**) with reduced duration of supported sitting and head control. This decline could not be objectified using motor scores. Chewing, swallowing, and respiration became increasingly challenging. Add-on therapy with risdiplam was initiated with the objective of improving endurance. Subsequently, parents observed a prolonged duration of supported sitting and a louder voice. 28 months after the initiation of add-on risdiplam, the girl achieved the ability of independent sitting with consistent head control.

***Patient 2***

Under intrathecal nusinersen treatment the boy learned to sit independently but not to crawl, stand or sit up and required an insufflator/exsufflator in addition to non-invasive ventilation. After switching to OA, his weight increased. Over time, he failed to thrive again, developed a progressive scoliosis and was admitted repeatedly for pneumonia. At the age of seven, videofluoroscopic swallow study confirmed aspirations during oral feeding. Add-on risdiplam was started to improve respiration and dysphagia, and a gastrostomy was performed due to aspirations and failure to thrive. He had no more pneumonia, and parents reported improved swallowing of liquids. In addition to weight gain, parents noted that the boy was more energetic and more stable at sitting. As the gastrostomy was inserted shortly after risdiplam, it is no longer feasible to utilize weight-for-age z-scores to evaluate dysphagia in this child.

***Patient 3***

The boy achieved free sitting at 24 months but did not acquire any further motor milestones. After a pneumonia, his weight dropped significantly, and his motor abilities decreased. Due to nocturnal hypoventilation in a sleep study and failure to thrive, nocturnal noninvasive ventilation, an insufflator/exsufflator and a gastrostomy were installed at 36 months of age. Add-on risdiplam was commenced to improve dysphagia and respiratory function. Parents noted an increase in muscle strength with improved head control and endurance as well as stronger coughing. He was admitted once for pneumonia during the 12 months follow-up.

***Patient 4***

The boy developed new motor skills after OA but then exhibited a plateau. At the age of 18 months, following a severe influenza H1N1 pneumonia with pneumothorax and mediastinal emphysema, requiring transient invasive ventilation, he lost the ability to sit without support, and required permanent non-invasive ventilation. Following a second pneumonia, the boy was no longer able to bring his feet to his mouth in supine, and non-invasive ventilation had to be extended to 21 hours per day. Add-on therapy with risdiplam was initiated with the objective of improving motor and respiratory function. The boy continuously gained weight and acquired the ability to sit independently. Non-invasive ventilation could be reduced to nocturnal-only. Following the initiation of add-on therapy with risdiplam, the boy experienced two additional instances of pneumonia during the 26-month follow-up period, necessitating inpatient treatment for a duration of six days per instance.

***Patient 5***

This boy had pneumonia at seven weeks, four and five months of age. Nocturnal non-invasive ventilation and gastrostomy were installed due to nocturnal hypoventilation in polysomnography, and repetitive aspirations, respectively. Add-on therapy with risdiplam was initiated with the objective of reducing hospitalizations. Following an additional pneumonia at eight months, invasive ventilation via tracheostomy was started. He received oral feeding only from 11 months of age when the speech therapist deemed this safe. At 19 months he had another pneumonia not requiring hospitalization. The boy achieved the ability to sit with the support of a spinal orthosis.
